# Eradication of Resistant and Susceptible Aerobic Gram-Negative Bacteria From the Digestive Tract in Critically Ill Patients; an Observational Cohort Study

**DOI:** 10.3389/fmicb.2021.779805

**Published:** 2022-02-03

**Authors:** Sophie H. Buitinck, Rogier Jansen, Rob J. Bosman, Nardo J. M. van der Meer, Peter H. J. van der Voort

**Affiliations:** ^1^Department of Intensive Care, OLVG Hospital, Amsterdam, Netherlands; ^2^TIAS School for Business and Society, Tilburg, Netherlands; ^3^Department of Medical Microbiology, OLVG Hospital, Amsterdam, Netherlands; ^4^Department of Critical Care Medicine, University Medical Center Groningen, University of Groningen, Groningen, Netherlands

**Keywords:** selective digestive tract decontamination, SDD, resistance, decontamination, critically ill, ICU

## Abstract

**Background:**

Selective Decontamination of the Digestive tract (SDD) aims to prevent nosocomial infections, by eradication of potentially pathogenic micro-organisms from the digestive tract.

**Objectives:**

To estimate the rate of and the time to eradication of resistant vs. susceptible facultative aerobic gram-negative bacteria (AGNB) in patients treated with SDD.

**Methods:**

This observational and retrospective study included patients admitted to the ICU between January 2001 and August 2017. Patients were included when treated with SDD (tobramycin, polymyxin B, and amphotericin B) and colonized in the upper or lower gastro-intestinal (GI) tract with at least one AGNB present on admission. Decontamination was determined after the first negative set of cultures (rectal and throat). An additional analysis was performed of two consecutive negative cultures.

**Results:**

Of the 281 susceptible AGNB in the throat and 1,087 in the rectum on admission, 97.9 and 93.7%, respectively, of these microorganisms were successfully eradicated. In the upper GI-tract no differences in eradication rates were found between susceptible and resistant microorganisms. However, the median duration until eradication was significantly longer for aminoglycosides resistant vs. susceptible microorganisms (5 vs. 4 days, *p* < 0.01). In the lower GI-tract, differences in eradication rates between susceptible and resistant microorganisms were found for cephalosporins (90.0 vs. 95.6%), aminoglycosides (84.4 vs. 95.5%) and ciprofloxacin (90.0 vs. 95.2%). Differences in median duration until eradication between susceptible and resistant microorganisms were found for aminoglycosides and ciprofloxacin (both 5 days vs. 6 days, *p* = 0.001). Decontamination defined as two negative cultures was achieved in a lower rate (77–98% for the upper GI tract and 64–77% for the lower GI tract) and a median of 1 day later.

**Conclusion:**

The vast majority of both susceptible and resistant microorganisms are effectively eradicated from the upper and lower GI tract. In the lower GI tract decontamination rates of susceptible microorganisms are significantly higher and achieved in a shorter time period compared to resistant strains.

## Introduction

Selective Digestive Decontamination (SDD) aims to prevent secondary infection by eradication of potentially pathogenic micro-organisms (PPM’s) from the respiratory and digestive tract (van Saene et al., 2003). This intervention has been studied in more than 70 RCTs and has been proven to be effective in infection prevention and mortality reduction (Silvestri et al., 2007; Silvestri and van Saene, 2012; Plantinga et al., 2018). Despite these results, SDD is still debated. The main concern is the emergence or selection of resistant micro-organisms when applying SDD (Cuthbertson et al., 2013).

In a systematic review of 35 studies on SDD and selective oral decontamination (SOD) the emergence of resistant strains was studied (Daneman et al., 2013). No association between the use of SDD and colonization or infection with resistant micro-organisms in ICU-patients was found. Some studies reported even a decline in the prevalence and incidence of antimicrobial resistant micro-organisms in respiratory and digestive tract cultures when applying SDD (Ochoa-Ardila et al., 2011; Smet et al., 2011; Houben et al., 2014; Wittekamp et al., 2015; Sanchez-Ramirez et al., 2018). The longest follow-up of 21 years continuous use of SDD confirmed that the fear for increased development of resistance could not be confirmed (Buitinck et al., 2019).

Moreover, in some studies application of SDD was initiated to eradicate resistant microorganisms from the gut. Oostdijk et al. (2012) studied the eradication rates of cephalosporin-resistant and cephalosporin-susceptible enterobacteriaceae and found that 73% of patients colonized with cephalosporin-resistant enterobacteriaceae were successful eradicated before ICU-discharge. In patients colonized with cephalosporin- susceptible enterobacteriaceae successful decolonization was reached in 80% (*p* = 0.17) (Oostdijk et al., 2012). For aminoglycoside-resistance, this percentage was 62% in resistant bacteria and 81% in susceptible bacteria, respectively (*P* < 0.01). Already in 1987, Stoutenbeek et al. (1987) reported that in almost all patients colonized with cefotaxime-resistant Gram-negative bacilli successful decolonization was accomplished within 1 week.

Despite these reports, the evidence on the eradication of resistant bacteria with the administration of SDD is scarce and its efficacy is not yet clear. The objective of this observational retrospective cohort is therefore to compare the rate and timing of successful decolonization for susceptible and resistant potentially pathogenic aerobic gram-negative bacteria (AGNB) from both upper and lower digestive tract in ICU-patients treated with SDD.

## Materials and Methods

### Study Design

This is a retrospective cohort analysis of microbiology data from all consecutive ICU patients admitted between January 2001 and August 2017. This study is conducted in a Dutch 20-bed adult mixed medical, surgical and cardiac surgery tertiary intensive care unit in an inner-city teaching hospital in Amsterdam. In this ICU, SDD is implemented in 1986 and has been used consistently, unchanged and without interruption. The local medical ethical review board (ACWO OLVG) approved the study and waived informed consent due to its retrospective and observational design in accordance to Dutch and European legislation (study no. WO 18.017).

### Patients

Patients were eligible for analysis when they had a primary carrier state with one or more potentially pathogenic *Enterobacteriaceae* (*Citrobacter* sp., *Enterobacter* sp., *E* coli, *Klebsiella* sp., *Morganella* sp., *Proteus* sp., *Serratia* sp.) or gram-negative non-fermenters (*Acinetobacter* sp., *Pseudomonas* sp.) in the upper or lower gastro-intestinal tract in the first surveillance cultures in the ICU irrespective of the number of colony-forming units. In addition, at least two follow up surveillance cultures taken during ICU-admission (cultures drawn on two different days) should be available and patients must have been treated with SDD during ICU-admission. Data on baseline characteristics of all included patients were prospectively recorded in the ICU database and extracted for this analysis. This data includes sex, age, APACHE IV predicted mortality, length of stay, patient category, date of admission, ICU-mortality, and antimicrobial treatment, both intravenously and topically applied. The general policy in this ICU is to promote defecation with laxatives from the second day of ICU admission onward to achieve defecation within 4 days.

### Cultures

Data on surveillance cultures taken during ICU-admission were extracted from the hospital database. Cultures of the throat were performed to determine carrier state in the upper gastro-intestinal tract. For the lower tract rectal cultures were performed. Routinely, cultures from throat and rectum were taken twice a week and tracheal aspirate 3 times a week for surveillance in patients treated with SDD. All culture samples taken in the context of SDD surveillance were plated on an unselective blood agar and four specific agars selecting for gram-positive bacteria, gram-negative bacteria, yeast and vancomycin resistant enterococci. AGNB’s were tested for antimicrobial susceptibility using agar disk-diffusion. These microorganisms were only included for further analysis if susceptibility testing to aminoglycosides (tobramycin or gentamicin), third generation cephalosporins, polymyxin and ciprofloxacin was performed. Strains were defined as resistant when they were tested intermediate or resistant, for at least one of the before mentioned groups. The cut off values for resistance were set following the guidelines of the “clinical and laboratory standards institute” (CLSI) until July 2011 and “the European committee on antimicrobial susceptibility testing” (EUCAST) from July 2011 until 2017 (Wayne, 2010; European Committee on Antimicrobial Susceptibility Testing (Eu-Cast), 2021). Susceptibility tests of cephalosporins were reported as measured, irrespective of potential presence of beta-lactamases such as AmpC beta-lactamase.

### Antimicrobial Treatment 2001–2017

Patients were treated with SDD when the expected ICU stay was more than 24 h, irrespective of the need for mechanical ventilation. This decision whether or not to start SDD was left to the discretion of the attending physician. According to the original SDD formulation, the SDD regimen consists of four times daily Orabase^®^, a sticky oral paste enriched with 2% polymyxin B, amphotericin B and tobramycin. In addition, 10 ml of a suspension containing 500 mg amphotericin B, 100 mg polymyxin B and 80 mg tobramycin is administered four times daily in the gastric tube or swallowed in patients without gastric tube. An i.v. course of cefotaxime is administered to all patients for 4 days but is prolonged in case of active infection with susceptible microorganisms or replaced by another antimicrobial agent in case of infection with a cefotaxime resistant microorganism. The choice for polymyxin B instead of polymyxin E might imply a more effective therapy when given in the same dose (Evans et al., 1999).

To the discretion of the attending physician, empirical antimicrobial treatment on admission is extended with ciprofloxacin i.v. or tobramycin i.v. In case of peritonitis metronidazole is added as well. Other i.v. antimicrobials can be given when previous culture results necessitate another choice. Penicillins are carefully avoided whenever possible due to their negative effects on the indigenous aerobic and anaerobic intestinal flora which could lead to a loss of the protective effect to invading pathogens (colonization resistance) (Vollaard, 1991). When the surveillance cultures showed Enterobacteriaceae with a combined polymyxin and tobramycin resistance, co-trimoxazole 2% was added in the oral paste and twice daily 960 mg in the enteral suspension until decontamination was obtained.

### Data-Analysis and Statistics

**FIGURE 1:**
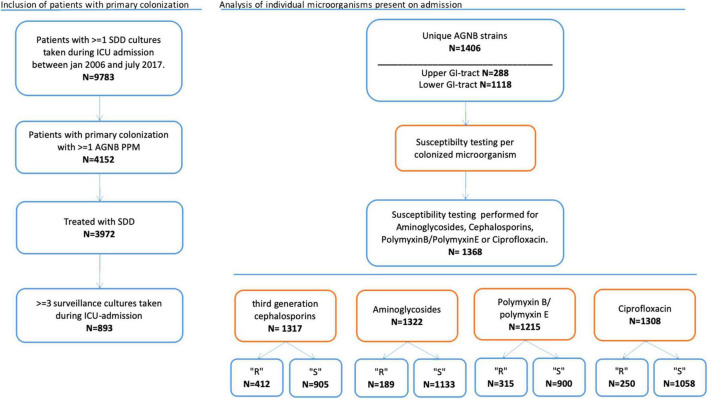
Flowchart of patients and study procedures.

The microorganisms cultured on admission were categorized in resistant and susceptible microorganisms per agent. Analysis was performed for all unique strains of AGNB found in the upper or lower GI tract. We analyzed the anti-microbial agents separately: third generation cephalosporins, aminoglycosides, polymyxin B/E and ciprofloxacin (Figure 1). Depending on whether susceptibility testing for the different agents was performed, microorganisms were included for each particular analysis. This implicates that most cultured microorganisms were included in more than one analysis.

The primary outcome was successful decontamination. Decontamination rates are reported as the percentage of AGNB’s present on admission that are successfully decontaminated from either the upper- or lower gastrointestinal tract. We have performed two analyses: (1) decontamination is achieved as soon as one follow-up culture is negative. (2) decontamination is achieved when two consecutive follow-up cultures are negative. Patients with one negative culture followed by ICU discharge were in this analysis considered as not-decontaminated. Differences in decontamination rates between susceptible and resistant micro-organisms are tested using Chi-square test.

The secondary outcomes of this study are the time to achieve successful decontamination and the rate of decontamination in case of co-resistance. Time to successful decontamination was given in days. Time to decontamination between susceptible and resistant strains was tested with the Mann-Whitney *U*-test (Table 1). The distributions of time to successful decontamination was estimated using the Kaplan Meier analysis and tested with the log-rank test.

**TABLE 1:** Mean and median duration until decontamination in days.

	Cephalosporins		Aminoglycosides		Colistin/Polymyxin B		Ciprofloxacin	
	Resistant	Susceptible	Resistant	Susceptible	Resistant	Susceptible	Resistant	Susceptible
**Upper GI-tract**								
Mean duration until eradication	5.1	4.2	5.9	4.5	4.9	4.5	5.3	4.5
Median duration until eradication	4.0	4.0	5.0	4.0	4.0	4.0	4.0	4.0
Standard error	0.19	0.15	0.45	0.12	0.20	0.16	0.40	0.12
	*P* = 0.14		*p* = 0.03		*p* = 0.21		*p* = 0.63	
**Lower GI-tract**								
Mean duration until eradication	6.6	5.9	7.7	5.7	5.6	6.1	5.7	7.4
Median duration until eradication	5.0	5.0	6.0	5.0	5.0	5.0	6.0	5.0
Standard error	0.21	0.11	0.42	0.10	0.19	0.12	0.27	0.10
	*p* = 0.001		*p* = 0.001		*P* = 0.015		*P* = 0.001	

## Results

### Patients and Cultures

A total of 867 patients with a primary carrier state with one or more aerobic Gram-negative microorganisms and treated with SDD were included. This accounts for 893 admissions (re-admissions included). In Table 2 baseline characteristics of the included admissions are summarized and shows that it is a mixed medical and surgical group of ICU patients with high APACHE scores. SDD was started on admission day 1 or 2 in 97.8% of patients. Patients included in the study were colonized with 288 unique AGNB’s in the upper gastro-intestinal tract (throat culture) of which 281 had antimicrobial sensitivity tested. In the lower gastro-intestinal tract (rectal culture) 1,118 unique AGNB’s were found of which 1,087 had susceptibility tested. The microorganisms cultured on admission in the upper gastro-intestinal tract were: *Acinetobacter* sp. (*N* = 21), *Citrobacter* sp. (*N* = 10), *Enterobacter* sp. (*N* = 23), *E. coli* (*N* = 39), *Klebsiella* sp. (*N* = 22), *Morganella* sp. (*N* = 9), *Proteus* sp. (*N* = 31), *Pseudomonas* sp. (*N* = 84) and *Serratia* sp. (*N* = 49). Unique strains of aerobic Gram-negative microorganisms in the lower GI-tract were: *Acinetobacter* sp. (*N* = 10), *Citrobacter* sp. (*N* = 36), *Enterobacter* sp. (*N* = 50), *E. coli* (*N* = 446), *Klebsiella* sp. (*N* = 70), *Morganella* sp. (*n* = 44), *Proteus* sp. (*N* = 161), *Pseudomonas* sp. (*N* = 283), and *Serratia* sp. (*N* = 18). Susceptibility testing was performed for third generation cephalosporins in 1,317 cases and for aminoglycosides in 1,322; for colistin in 1,215 cases and for ciprofloxacin in 1,308 cases respectively.

**TABLE 2:** Baseline characteristics.

Number of patients included		867	
Number of admissions		893	
Age in years, mean (SD)		69	(15)
Males, *N* (%)/Females *N* (%)		573 (64.2%)/320 (35.8%)	
Length of ICU stay in days, median (IQR)		12	(6.5–17.5)
APACHE IV predicted mortality		0.42	(0.18–0.67)
Readmission, *N* (%)		66	(11.8%)
Patient category	Cardiothoracic surgery, N (%)	198	(22.2%)
	Internal medicine, *N* (%)	170	(19.0%)
	Surgery, *N* (%)	233	(26.1%)
	Cardiology, *N* (%)	125	(14.0%)
	Pulmonology, *N* (%)	119	(13.3%)
	Neurology, *N* (%)	28	(3.1%)
	other, *N* (%)	20	(2.2%)
Mechanically ventilated (*N*,%)	706		(81.4%)
ICU mortality, *N* (%)		175	(19.6%)
Number of days treated with SDD, median (IQR)		12	(6.5–17.5)
SDD started on day 1 or day 2, *N* (%)		874	(97.8%)

### Decontamination Defined as One Follow-Up Culture Negative for Aerobic Gram-Negative Bacteria

Successful decontamination of aerobic Gram-negative microorganisms cultured on admission in the upper GI-tract was achieved for 275 out of 281 unique (susceptibility tested) strains (97.9%) before discharge. In the lower GI-tract the number of successfully decontaminated strains was 1,019 out of 1,087 unique strains (93.7%). Table 3 shows the success rates for decontamination in resistant and susceptible microorganisms for primary carrier state of the upper GI-tract and lower GI-tract separately. Decontamination appears to be achieved in over 90% for all cases except for aminoglycosides resistant strains in the lower GI tract (84%). The upper GI tract did not show significant differences in decontamination rates between susceptible strains and strains resistant for a specific antibiotic. Decontamination of the lower GI tract was significantly less successful in strains resistant to cephalosporins, aminoglycosides and ciprofloxacin compared to susceptible strains.

**TABLE 3:** Rates of successful decontamination according to location and antimicrobial agent, for resistant and sensitive strains.

Antimicrobial agent tested		Total number of colonization on admission with susceptibility testing.	Successful de-colonization, N	% Successful de-contamination	
**Upper GI-tract**					
Cephalosporins	R	121	116	95.9%	*p* = 0.07
	S	150	149	99.3%	
Aminoglycosides	R	35	33	94.3%	*p* = 0.17
	S	242	238	98.3%	
Colistin	R	93	90	96.8%	*p* = 0.07
	S	163	160	98.2%	
Ciprofloxacin	R	40	38	95.0%	*p* = 0.21
	S	233	229	98.3%	
**Lower GI-tract**					
Cephalosporins	R	291	262	90.0%	*p* < 0.01
	S	755	722	95.6%	
Aminoglycosides	R	154	130	84.4%	*p* < 0.01
	S	891	851	95.5%	
Colistin	R	222	211	95.0%	*p* = 0.35
	S	737	693	94.0%	
Ciprofloxacin	R	210	189	90.0%	*p* < 0.01
	S	825	785	95.2%	

Table 4 show the decontamination rates for micro-organisms with co-resistance for two or more antimicrobial agents for the upper and lower GI tract, respectively. These rates are 95% or higher in both resistant and sensitive strains in the upper GI tract. In the lower GI tract co-resistant strains are significantly less often decontaminated when co-resistance is present.

**TABLE 4:** Decontamination rates for antimicrobial agents with co-resistance in the upper and lower gastrointestinal (GI) tract.

		*N*	Successful decolonization *N*	%	*p*-value
**Upper GI-tract**					
Co-resistance for at least two agents	Yes	75	71	94.7%	0.04
	No	206	204	99.0%	
Co-resistance for tobramycin and cefalosporins	Yes	24	22	91.7%	0.08
	No	257	253	98.4%	
Co-resistance for tobramycin and colistin	Yes	43	41	95.3%	0.23
	No	238	234	98.3%	
Co-resistance for tobramycin, colistin and cefalosporins.	Yes	7	7	100.0%	1.00
	No	274	268	97.8%	
**Lower GI tract**					
Co-resistance for at least two agents	Yes	219	189	86.3%	<0.01
	No	868	830	95.6%	
Co-resistance for tobramycin and cefalosporins	Yes	93	75	80.6%	<0.01
	No	994	944	95.0%	
Co-resistance for tobramycin and colistin	Yes	56	48	85.7%	0.02
	No	1,031	971	94.2%	
Co-resistance for tobramycin, colistin and cefalosporins	Yes	12	8	66.7%	<0.01
	No	1,075	1,011	94.0%	

The decontamination rates for specific microorganisms are summarized in Supplementary Table 1 and shows successful decontamination in more than 90% for all species except for Morganella (79%).

**FIGURE 2:**
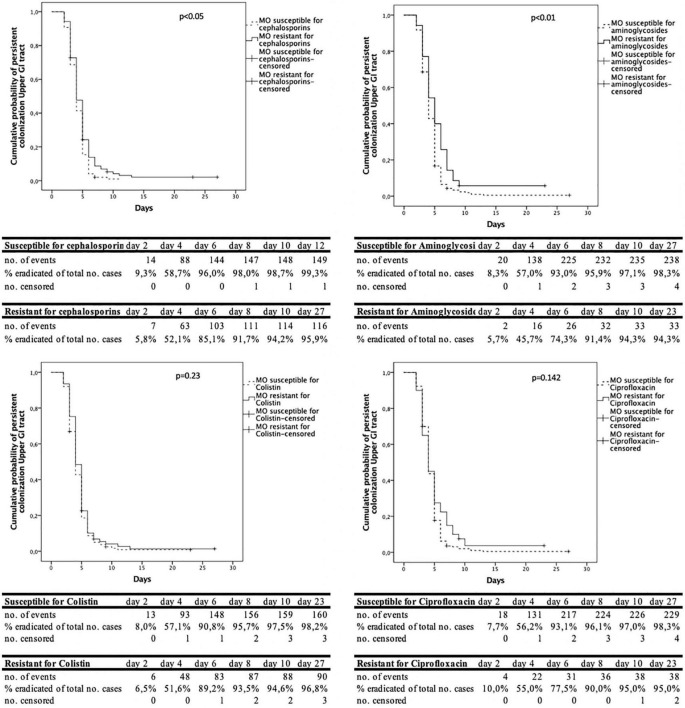
Cumulative decontamination over time for susceptible and resistant microorganisms for the upper gastrointestinal (GI) tract.

**FIGURE 3:**
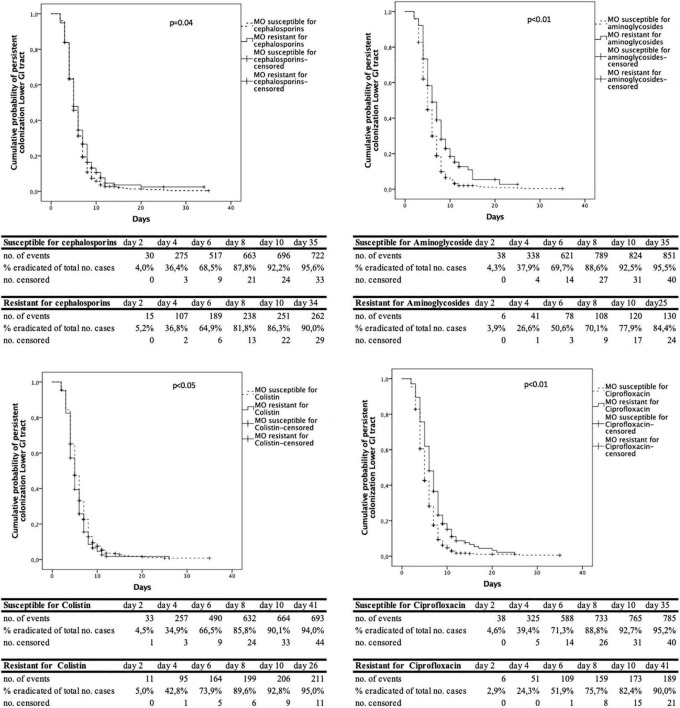
Cumulative decontamination over time for susceptible and resistant microorganisms for the lower gastrointestinal (GI) tract.

The analysis of the time to decontamination was performed for colonization of the upper and lower GI-tract separately. Figure 2 shows the Kaplan-Meyer curves for the different antimicrobial agents tested in the upper GI tract. The same information in shown in Figure 3 for the lower GI-tract.

The time to successful decontamination appears to be significantly longer for resistant microorganisms compared to sensitive microorganisms (Table 1).

### Decontamination Defined as Two Consecutive Follow-Up Cultures Negative for Aerobic Gram-Negative Bacteria

We also analyzed decontamination defined as two negative follow-up cultures before ICU discharge. For susceptible strains the rates were between 91.4 and 98.0% in the upper gastrointestinal tract and between 73.7 and 76.6% in the lower gastrointestinal tract. For resistant strains the decontamination rates were between 77.1 and 90.3% in the upper gastrointestinal tract and between 61.7 and 76.6% for resistant strains in the lower gastrointestinal tract (Table 5). The median time to decontamination of the upper GI tract was 4 days for susceptible strains and 5 days for resistant strains except for colistin it was 4 days in both susceptible and resistant groups. The median time to decontamination of the lower GI tract was 5 days for susceptible and 8 days for ciprofloxacin resistant strains, 6 vs. 8 days for aminoglycosides resistant strains, 6 days for both cephalosporins susceptible and resistant strains and 4 days for both colistin susceptible and resistant strains.

**TABLE 5:** Decontamination rates when decontamination is defined as two consecutive negative cultures.

Antimicrobial agent tested		Total number of colonization on admission with susceptibility testing.	Successful de-colonization, N	Successful de-contamination %	*p*-value
**Upper GI-tract**					
Cephalosporins	R	121	103	85.1%	*p* < 0.01
	S	150	147	98.0%	
Aminoglycosides	R	35	27	77.1%	*p* < 0.01
	S	242	227	93.8%	
Colistin	R	93	84	90.3%	*p* = 0.47
	S	163	149	91.4%	
Ciprofloxacin	R	40	31	77.5%	*p* < 0.01
	S	233	220	94.4%	
**Lower GI-tract**					
Cephalosporins	R	291	196	67.4%	*p* < 0.01
	S	755	576	76.3%	
Aminoglycosides	R	154	95	61.7%	*p* < 0.01
	S	891	677	76.0%	
Colistin	R	222	170	76.6%	*p* = 0.39
	S	737	543	73.7%	
Ciprofloxacin	R	210	135	64.3%	*p* < 0.01
	S	825	632	76.6%	

The cumulative proportion of decontamination and Kaplan-Meier curves for susceptible and resistant strains are shown in Supplementary Figures 1, 2.

## Discussion

We have shown that high rates of decontamination in both susceptible and resistant microorganisms are achieved. SDD resulted in an overall level of decontamination in the upper GI tract of 97.9, and 93.7% in the lower GI tract before discharge from the ICU. We found significant differences in decontamination rates of the lower GI tract between susceptible and resistant micro-organisms for all antimicrobial agents, except for colistin, probably because of the high concentrations in the gut lumen. These findings demonstrate that many susceptible and resistant microorganisms can be decontaminated from the gut with SDD. The decontamination rate was lower when co-resistance was present. This finding was significant for microorganisms present in the lower GI-tract. Nonetheless, decontamination of the lower GI-tract was successful in 80.6% of microorganisms with co-resistance to tobramycin and cefalosporins, 85.7% of microorganisms with co-resistance to tobramycin and colistin; and 66.7% of microorganisms with combined resistant to tobramycin, colistin and cefalosporins. Decontamination rates in the presence of co-resistance was even higher in microorganisms found in the upper GI-tract. So, despite of co-resistance, decontamination was still possible in the majority of these microorganisms. It should be emphasized that successful decontamination is important to achieve the goals of SDD, the prevention of secondary bacterial infections in particular pneumonia and bacteremia. When decontamination is not achieved, the successful prevention of these infections will diminish.

In this study, when resistance to third generation cephalosporins, aminoglycosides or ciprofloxacin was present the duration until successful decontamination was longer compared to susceptible microorganisms. The Kaplan- Meier curves show that most of the micro-organisms are decontaminated in the first 6 days, both for resistant and susceptible micro-organisms. After day 8–10 after admission, much less cases were successfully decontaminated. We hypothesize that this may be due to the high concentrations of tobramycin and polymyxin, which exceed high MIC values, resulting in high elimination rates of (co) resistant and susceptible microorganisms (Vollaard, 1991). New resistant microorganisms during SDD treatment rarely appear, as was shown by our group previously (Buitinck et al., 2019).

The few studies on decontamination of Gram-negative microorganisms that have been performed show similar decontamination rates. Oostdijk et al. (2012) found decontamination rates of 62–81% with the lowest rates for aminoglycoside resistant Enterobacteriaceae. They defined decontamination as two consecutive negative cultures. Our decontamination rates with two consecutive negative cultures are reported as well and figures and tables as Supplementary Material. Table 5 shows similar or higher rates as Oostdijk et al. (2012). This is, however, an underreporting as patients with only one negative follow-up culture and subsequent ICU discharge are considered not-decontaminated while a prolonged stay in the ICU might show a second negative culture and thus successful decontamination. Therefore, our primary outcome measure was one negative culture. The one-negative culture rates are slightly overestimating as occasionally a patient is tested positive again in the next culture. Stoutenbeek et al. (1987) reported in cephalosporin resistant enterobacteriaceae an elimination rate of 82%. In contrast, Abecasis et al. (2011) in pediatric patients showed a much lower rate of 54% in ESBL-producing AGNB’s. In this study AGNB-ESBL susceptible for tobramycin and AGNB-ESBL resistant to tobramycin were also analyzed separately. In the tobramycin susceptible group, no failures of clearance were reported, whereas in the tobramycin resistant group a failure rate of 39% was reported.

The fact that not all patients are successfully decontaminated stresses the importance of surveillance cultures and a pro-active approach when decontamination is not reached a week after admission. The reasons for decontamination failure may be a slow transit time or ileus that prevents the substances to reach the rectal cavity. In our unit we have a general policy to achieve defecation within 4 days by using laxatives. This may be an explanation, next to the definition of one instead of two cultures, for the relatively fast decontamination time in comparison with other studies. Our analysis for two consecutive cultures also shows a faster decontamination than other studies (Supplementary Figures 1, 2). When the throat is not successfully decontaminated, *foreign bodies*, e.g., a nasogastric tube that is in place for more than a week, may maintain pathological colonization (Van Der Voort et al., 2019). In addition, modification of the components of SDD, e.g., the addition of co-trimoxazole or amikacin, could lead to decontamination in specific cases (Nahar et al., 2019; Van Der Voort et al., 2019).

The present study has several strengths and limitations. First, in this cohort study the protocol of SDD is consequently applied in patients with expected ICU stay of more than 24 h irrespective of the need for mechanical ventilation over the complete 10-year study period. SDD was started in 97.8% of patients on admission day 1 or admission day 2. Second, surveillance cultures were consistently taken twice weekly. Third, only surveillance cultures were used for the determination of successful decontamination instead of organ site cultures. SDD surveillance cultures have been shown in previous research to have a greater sensitivity for culturing potentially pathogenic microorganisms than organ sites (Viviani et al., 2010).

This study has several limitations too. The observational design limits the possibility for correction of confounders. A previously determined factor in studies that influences the rate of decontamination is ileus and gastroparesis. These situations limit the propulsion of enteral antimicrobial agents through the gut and therefore limit the success rate of decontamination. We could not reliably determine from our database which patients suffered from ileus or gastroparesis and who did not. On the other hand, our study describes the real-life situation and daily practice.

In practice, successful decontamination is usually determined after two negative cultures. In this study we analyzed one and also two consecutive negative cultures as a definition of decontamination. Two negative cultures give a fair amount of underreporting as quite a number of patients are discharged after one negative culture and are, in that case, counted as not-decontaminated. We have shown (Table 5 and Supplementary Figures 1, 2) that the rectal cultures show lower decontamination rates when defined as two negative cultures and also a slower decontamination compared to the results of one negative culture (median 5 days for the upper GI tract vs. 4 days, and 6 days for the lower GI tract vs. 5 days). It is emphasized that a first negative culture implies a great reduction in the number of AGNB in this patient, which will probably reduce both transmission and secondary infection. In addition, this approach was chosen to include more patients as most patients were discharged within a week, the time needed to have three consecutive cultures. Defining decontamination as one negative culture leads to somewhat higher decontamination rates compared to the decontamination rates for two consecutive negative cultures which might be a slight overestimation of the decontamination rate in case a follow-up culture becomes positive again.

A statistical limitation is in the Kaplan-Meier survival analysis as one of the assumptions for this analysis is that the event is precisely measured. In practice, surveillance cultures are taken on admission and twice weekly. The exact date of decontamination could not be determined because of the 3–4-day interval between cultures. Interval censoring may lead to false positive results (Rucker and Messerer, 1988). Also, due to differences in censoring patterns, the result of log-rank testing could be partly the effect of differences in censoring than differences in probability distributions. However, when looking at the Kaplan Meier curves, not only a difference in censoring, but also a difference in time to decontamination can be seen. We used the Kaplan Meier curves more as a tool to show the dynamics in decontamination than as a tool to proof differences between groups.

The interval between the cultures might also have caused an underestimation of the rate of successful decontamination. Patients who were discharged some days after a positive culture may have been decontaminated at the time of discharge without being recognized as such because of the absence of new cultures at discharge. Last, it is unknown whether rebound colonization occurs after ICU discharge.

This study shows that in most, but not all patients, aerobic Gram-negative microorganisms are eliminated from the gut in critically ill patients during SDD treatment. Moreover, when decontamination is not achieved but the growth density in the cultures is decreased than the risk for secondary infection and cross-contamination is probably reduced as infection usually occurs after a state of overgrowth (van Saene et al., 2003). Future research might focus on the reasons why some patients experience prolonged and abnormal carrier state. In addition, the use of alternative antimicrobials can be studied to their efficacy to decontaminate.

## Conclusion

In conclusion, we have shown that most susceptible and resistant microorganisms can be cleared from the gut. However, microorganisms that are resistant to aminoglycosides, cephalosporins or ciprofloxacin are less frequently eradicated and the duration until decontamination is prolonged.

## Data Availability Statement

The raw data supporting the conclusions of this article will be made available by the authors, without undue reservation.

## Ethics Statement

The studies involving human participants were reviewed and approved by ACWO. Written informed consent for participation was not required for this study in accordance with the national legislation and the institutional requirements.

## Author Contributions

SB analyzed the data and drafted the manuscript. PV, SB, and RJ designed the study. RJ gave access to the microbiology data. All authors were involved in writing the manuscript.

## Conflict of Interest

The authors declare that the research was conducted in the absence of any commercial or financial relationships that could be construed as a potential conflict of interest.

## Publisher’s Note

All claims expressed in this article are solely those of the authors and do not necessarily represent those of their affiliated organizations, or those of the publisher, the editors and the reviewers. Any product that may be evaluated in this article, or claim that may be made by its manufacturer, is not guaranteed or endorsed by the publisher.

## Supplementary Material

Supplementary Figure 1 The cumulative proportion of decontamination and Kaplan-Meier curves for susceptible and resistant strains in the upper gastrointestinal tract.

Supplementary Figure 2 The cumulative proportion of decontamination and Kaplan-Meier curves for susceptible and resistant strains in the lower gastrointestinal tract.

Supplementary Table 1 Decontamination rates per microorganism.

## Abbreviations

ACWO: Advies Commissie Wetenschappelijk Onderzoek
AGNB: Aerobic Gram Negative Bacteria
GI: gastro-intestinal
ICU: Intensive Care Unit
I.v.: intravenous
PPM: Potential Pathogenic Microorganism
RCT: Randomized Controlled Trial
SDD: Selective Digestive Tract Decontamination.
