# Supplementary figures and images for: Eradication of Resistant and Susceptible Aerobic Gram-Negative Bacteria From the Digestive Tract in Critically Ill Patients; an Observational Cohort Study
Source: Front Microbiol. 2022 Feb 3;12:779805. doi: 10.3389/fmicb.2021.779805 (PMC8853443; doi:10.3389/fmicb.2021.779805)

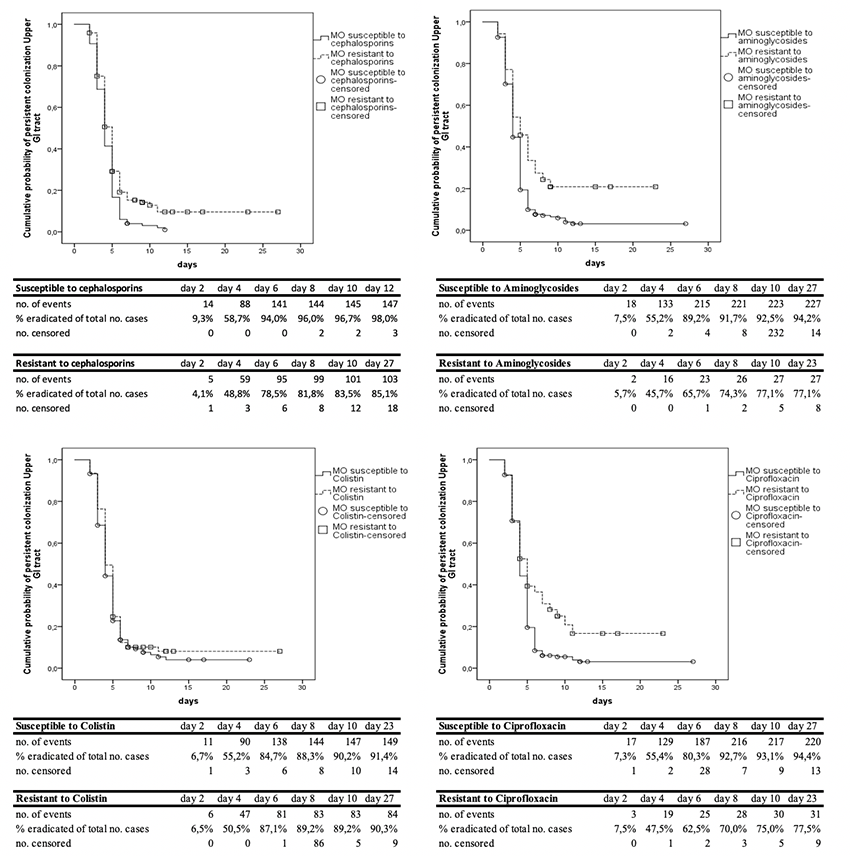

Supplement: Supplementary Figure 1 — The cumulative proportion of decontamination and Kaplan-Meier curves for susceptible and resistant strains in the upper gastrointestinal tract. [file Image_1.tiff]

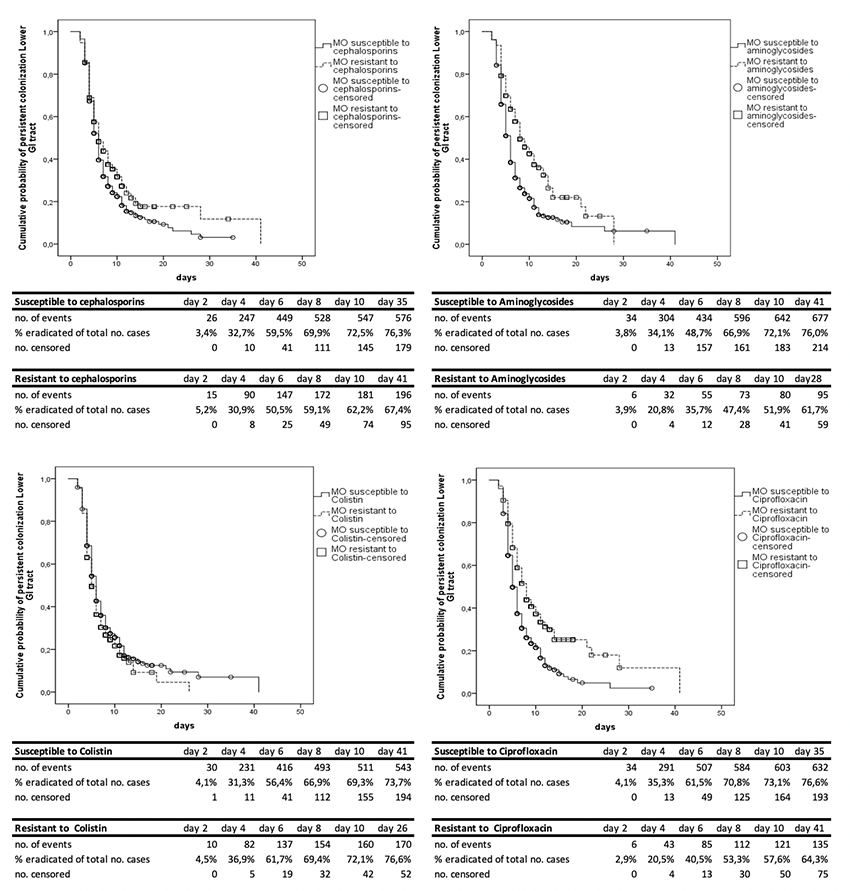

Supplement: Supplementary Figure 2 — The cumulative proportion of decontamination and Kaplan-Meier curves for susceptible and resistant strains in the lower gastrointestinal tract. [file Image_2.tiff]
