# Supplementary material for: Eradication of Resistant and Susceptible Aerobic Gram-Negative Bacteria From the Digestive Tract in Critically Ill Patients; an Observational Cohort Study
Source: Front Microbiol. 2022 Feb 3;12:779805. doi: 10.3389/fmicb.2021.779805 (PMC8853443; doi:10.3389/fmicb.2021.779805)
Supplement: Supplementary Table 1 — Decontamination rates per microorganism. [file Table_1.DOCX]

**Table S1. Decontamination rates per microorganism**

| Upper gastrointestinal tract | | | |
| --- | --- | --- | --- |
| Microorganism | Frequency | Successful  decontamination  before  discharge (N) | Successful  decontamination  before  discharge (%) |
| Acinetobacter | 20 | 19 | 95.0% |
| Citrobacter | 9 | 9 | 100.0% |
| Enterobacter | 23 | 23 | 100.0% |
| E. coli | 39 | 38 | 97.4% |
| Klebsiella | 22 | 21 | 95.5% |
| Morganella | 9 | 9 | 100.0%% |
| Proteus | 31 | 31 | 100.0% |
| Pseudomonas | 80 | 80 | 100.0% |
| Serratia | 48 | 45 | 93.8% |
|  |  |  |  |
| Lower gastrointestinal tract | | | |
| Microorganism | Frequency | Successful  decontamination  before  discharge (N) | Successful  decontamination  before  discharge (%) |
| Acinetobacter | 10 | 10 | 100.0% |
| Citrobacter | 36 | 34 | 94.4% |
| Enterobacter | 50 | 49 | 98.0% |
| E. coli | 436 | 411 | 94.3% |
| Klebsiella | 64 | 59 | 92.2% |
| Morganella | 43 | 34 | 79.1% |
| Proteus | 157 | 153 | 97.5% |
| Pseudomonas | 273 | 251 | 91.9% |
| Serratia | 18 | 18 | 100.0% |
